# Quadrivalent Live-Attenuated Influenza Vaccine in Milan preschools: an Italian experience of school-located flu vaccination within the 2022–2023 season

**DOI:** 10.1186/s13052-024-01649-2

**Published:** 2024-05-13

**Authors:** Stefania Roncaglia, Antonella Amendola, Paola Baldassarre, Francesca Barilli, Silvia Bianchi, Andrea Biuso, Claudia Chillemi, Clara Fappani, Chiara Gasparini, Maria Gori, Gabriella Guida, Francesca Ripoli, Laura Sgambetterra, Elisabetta Tanzi, Gian Vincenzo Zuccotti

**Affiliations:** 1 Department of Paediatrics, Buzzi Children’s Hospital, Milan, Italy; 2 Department of Health Sciences, Università Degli Studi Di Milano, Milan, Italy; 3 EpiSoMI CRC -Coordinated Research Center, Università Degli Studi Di Milano, Milan, Italy; 4 Department of Biomedical and Clinic Sciences, Università Degli Studi Di Milano, Milan, Italy

**Keywords:** Influenza, Children, Flu vaccination, Quadrivalent Live Attenuated Influenza Vaccine (qLAIV), Widespread immunization, School-located vaccination program

## Abstract

**Background:**

In Italy, since the 2020–2021 flu season, the flu vaccine recommendation was extended to all children aged 6 months to 6 years and quadrivalent Live-Attenuated Influenza Vaccine (qLAIV) was introduced. Since school-aged children are important carriers of annual influenza epidemics, a school-based influenza vaccination program may potentially increase vaccine uptake. Recent studies, conducted in the UK and the US, show that school-based vaccination can reach higher percentage of paediatric vaccination coverage compared to children vaccinated in other settings.

**Methods:**

During 2022–2023 flu season in 9 preschools located in Milan healthcare personnel vaccinated children with qLAIV at the end of a school day. A Google Form questionnaire was administered to preschoolers’ parents of all preschools within the Municipality of Milan.

**Results:**

In the preschools engaged in the vaccination program, 233 out of 1939 children were vaccinated (12%). Among these, 61 (26.2%) had never been vaccinated for influenza before. Vaccination coverage was 11.5% for Italian children and 14.3% for children coming from an immigrant background.

We collected 3659 questionnaire responses, divided according to study participation status (371 from preschools that participated in the vaccination program and 3288 from other preschools in Milan). 57% of the families who answered to the questionnaire vaccinated their children for flu. qLAIV accounted for 85.6% of vaccinations. We observed a statistically significant difference in the percentage of vaccinated children between those attending a school participating in the project (67.9%) and children attending other schools (56%) (*p* < 0.001). Vaccination was administered by family pediatricians (48.9%), in vaccination centers (34.8%), in vaccine hubs (11.3%), in schools (2.6%), by private pediatricians (1.6%) and in other settings (0.7%). Focusing on the responses from families whose children attend schools participating in the vaccination program, 21.8% stated that the vaccination was provided in school.

**Conclusion:**

According to our experience, in Italy, at the moment, only the cooperation between health providers and alternative settings, including schools, may expand flu vaccination coverage. In particular, schools are to be considered a place to inform and reach out to families, useful to increase vaccination coverage.

## Background

Influenza has a substantial impact on the pediatric population in Italy, As it affects 20–30% of paediatric population every year [1].

Historical data reveals a challenge in achieving adequate influenza vaccination coverage in children, particularly evident in the poor percentages observed until the 2019–2020 flu season, when a peak of 4.2% was reached in children aged 2 to 4 years [2].

In order to improve vaccination coverage, from the 2020–2021 flu season the Italian Ministry of Health extended vaccine recommendation to all children aged 6 months to 6 years [3]. In the 2020–21 flu season vaccination coverage increased for all ages of paediatric population (9.2% aged 6–23 months, 19% aged 2–4 years, 13.1% aged 5–8 years, 6% aged 9–14 years, 4.5% aged 15–17 years) [4]. This result has been basically maintained in the 2022–2023 flu season (7.2% 6–23 months, 9.2% 2–4 years, 22.6% 5–8 years, 4.9% 9–14 years, 2.1% 15–17 years) [5].

The increase of vaccination coverage in the 2020–2021 flu season also coincides with the introduction, for the first time in Italy, of quadrivalent Live-Attenuated Influenza Vaccine (qLAIV) in addition to the Inactivated Influenza Vaccine (IIV) [6]. The qLAIV is indicated for children and adolescents aged 24 months to 18 years, administered by intranasal route [7]. The qLAIV was first approved as a trivalent vaccine for children aged 2–18 years in 2011 [8]; since the 2014–15 flu season the qLAIV has been approved as the vaccine of choice for children in UK vaccination programme [9, 10].

The first Italian study of using the qLAIV on 9292 children aged 2–18 years, during the 2020–2021 flu season, has confirmed its safety and ease of administration and a high degree of satisfaction among both health workers and parents was recorded [11].

Assuming that school-aged children are important drivers of annual influenza epidemics, to further increase the vaccination coverage, a school-based influenza vaccination program may potentially increase vaccine uptake [11]. A pilot project was set up in the schools of England [12] and Scotland [13] during the 2013–2014 flu season reaching the highest percentage of paediatric vaccination coverage (~ 50%). Following successful pilots in primary schools in 2013–14, in the 2016–17 season a pilot project was developed in Wales to introduce qLAIV vaccination sessions in local nursery schools, then routinely it has been offered in subsequent years [14]. The workforce for the school-setting vaccination program in England consisted of qualified nurses and specific vaccination teams [15]. The school setting showed several clear advantages: be more familiar for children; no absence from school to be immunised; minimal disruption to the school timetable; parental attendance or involvement are not mandatory once informed consent has been provided [16]. Overall, school-based vaccination generally resulted higher vaccination attendance than General Practitioner practices [15].

In US Szilagyi PG et al. has shown effectiveness of school-located influenza vaccination [17, 18], suggesting a moderate impact registered in both elementary and secondary schools, reaching an increase of 5.8 and 5.5 percentage points, respectively, compared to children vaccinated “anywhere” [19].

During 2021–2022 flu season, a first experience in Italy of flu vaccination at school was carried out. This pilot study, conducted in 5 preschools in Milan, showed a good response of families to flu vaccination, also involving those with immigrant background. It seems that school could be the ideal place to overcome socioeconomic and cultural barriers [20].

The aim of this study is to confirm the possible role of the school as a flu vaccination provider and the feasibility of this setting to increase influenza vaccination coverage in the paediatric population.

## Methods

**Fig. 1:**
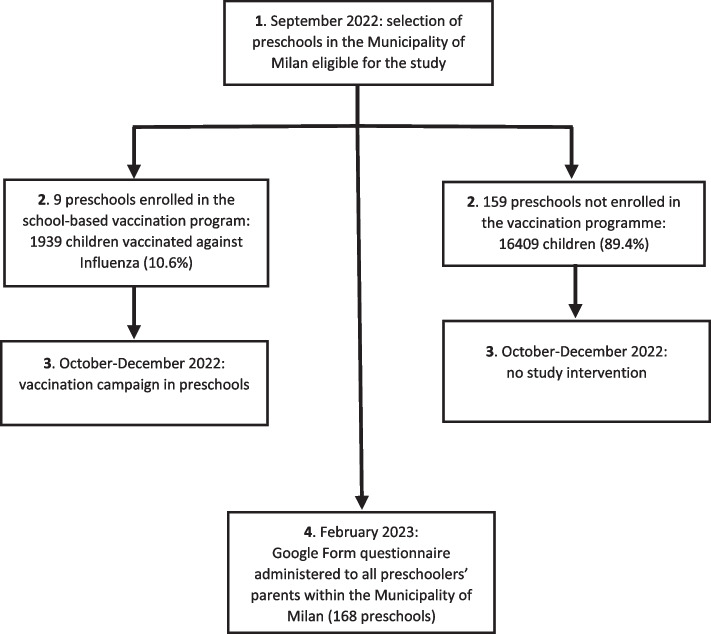
Study flow diagram

During 2022–2023 flu season collaborative partnership has been established among the Municipality of Milan, Buzzi Children's Hospital, the University of Milan and the Lombardy Region. All study documents, including informed consent form and protocol received approval by the Milano Area 1 Ethical Committee. The diagram of the study flow is shown by Fig. 1. The Municipality of Milan selected a sample of 9 preschools (children aged 3–5 years), choosing one from each zone of Milan. Dedicated webinars with informative material for families on influenza and flu vaccination were produced and distributed. In the preschools engaged in the vaccination program vaccination was offered at school. In other preschools, two vaccination Open Day were promoted in the weekend. A designated day was assigned to each school for the vaccination session. The Municipality of Milan's personnel collected parental participation in advance. On the designated vaccination day, between 10 November and 21 December 2022, starting from the end of the school day, children were accompanied by at least one parent to the vaccination venue. They brought along their informed consent for vaccination and completed an anamnesis form. The healthcare personnel, notably paediatric residents, verified the health information provided by parents regarding the child's health status and potential contraindications to vaccination. Healthy children were immunized with the qLAIV (Fluenz Tetra™ AstraZeneca Italy). After the vaccination, children were observed for 15 min and a vaccination certificate was issued. If the child received the flu vaccination for the first time, an appointment for the booster dose was scheduled at least 4 weeks later.

In February 2023 a Google Form questionnaire was administered to preschoolers’ parents of all 168 preschools within the Municipality of Milan. The questionnaire inquired whether the child received the influenza vaccine in 2022–2023 flu season and the context in which the vaccination was administered (Table 1).

**Table 1:** Questionnaire for parents

*Preventing the flu: flu vaccination in Milan preschool children:*	
**Name of the school your child attends:** _______________________________	**Did your child receive flu vaccination in** **the year 2022–2023?** ○ Yes ○ No
**Year of birth of your child:**	**If yes, which vaccine?**
○ 2016 ○2017 ○ 2018 ○ 2019 ○ 2020	○ Quadrivalent Live-Attenuated Influenza Vaccine (qLAIV) ○ Inactivated Influenza Vaccine (IIV)
**Sex**	**If yes, where?**
○ Male ○ Female	○ Family paediatrician ○ Private paediatrician ○ School ○ Vaccination center ○ Vaccination Hub ○ Others

Characteristics of population are described using numbers and percentages. Missing data are excluded when computing percentages.

We have applied the chi-square (χ2) test on the statistical analysis. A *p*-value < 0.05 is considered statistically significant. All analyses were performed using OpenEpi 3.01 software.

## Results

The total number of children enrolled in the preschool vaccination program was 1939, whereas the number of children enrolled in preschools not involved in the vaccination program was 16,409.

In the schools engaged in the vaccination program, the demographic breakdown revealed a near balance between females and males and class ages. Of these participants, 1576 (81.3%) held Italian nationality, while 363 (18.7%) were of foreign background. Examining the vaccination outcomes, 233 out of 1939 children (12%) were vaccinated. Among these, 61 (26.2%) had never been vaccinated for the flu. Vaccination coverage stood at 11.5% for Italian children (181/1576) and 14.3% for foreign children (52/363). Demographics and vaccination outcomes of children enrolled in the vaccination program are shown in Table 2.

**Table 2:** Demographics and vaccination outcomes of children enrolled in the vaccination program

Demographics	All enrolled	Vaccinated
**Sex**		
Females	954 (49.2%)	119 (12.5%)
Males	985 (50.8%)	114 (11.6%)
**Age**		
Age 2–4	1298 (66.9%)	164 (12.6%)
Age 5–6	641 (33.1%)	69 (10.8%)
**Background**		
Italian	1,576 (81.3%)	181 (11.5%)
Foreign	363 (18.7%)	52 (14.3%)
**Total**	1939 (100%)	**233 (12**%**)**

Examining the vaccination outcomes among different schools, a different participation rate was observed, ranging from 4.6% to 20.2% (Table 3).

**Table 3:** Vaccination outcomes among different preschools within the Municipality of Milan

School zone	All enrolled	All Vaccinated	New Vaccinated
Zone 1	206	32 (15.5%)	5 (15.6%)
Zone 2	293	27 (9.2%)	14 (51.8%)
Zone 3	289	23 (8.0%)	2 (8.7%)
Zone 4	206	22 (10.7%)	3 (13.6%)
Zone 5	161	25 (15.5%)	7 (28.0%)
Zone 6	163	33 (20.2%)	5 (15.1%)
Zone 7	193	24 (12.4%)	10 (41.7%)
Zone 8	240	11 (4.6%)	2 (18.2%)
Zone 9	188	36 (19.1%)	13 (36.1%)

We didn’t observe any immediate side effects during the vaccination sessions, anyway this study did not include monitoring of adverse events of the qLAIV.

**Fig. 2:**
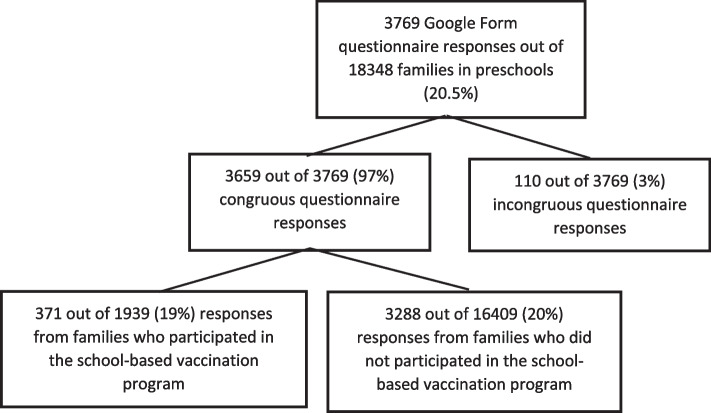
Google Form questionnaire responses

The Google Form questionnaire was filled by 20.5% (3769/18348) of all families of children who attend preschools within the Municipality of Milan. This led to a valid response count of 3659, equivalent to a response rate of 19.9% (3659/18348): 19% (371/1939) from those who participated in the school-based vaccination program and 20% (3288/16409) from those who did not (Fig. 2).

The type of vaccine predominantly administered was the qLAIV, accounting for 85.6% of vaccinations.

The 57% (2093/3659) of families who responded to the questionnaire vaccinated their children for flu. These answers were divided according to their study participation status, as shown in Table 4. We observed a statistically significant difference in the percentage of vaccinated children between those attending a school participating in the project (67.9%) and children attending other schools (56%) (*p* < 0.001) (Table 4).

**Table 4:** Vaccination results in each studied preschool

	**Vaccination status**			
		***Vaccinated***	***Unvaccinated***	***Total***
**Participation in the vaccination program at school**	***Yes***	252 (67.9%)	119 (32.1%)	371 (100%)
	***No***	1841 (56%)	1447 (44%)	3288 (100%)
	***Total***	2093	1566	3659

Concerning the vaccination providers, 48.9% of parents indicated the family pediatrician, 34.8% the vaccination center, 11.3% the vaccination hub, 2.6% the school, 1.6% a private pediatrician and 0.7% other. Focusing on the responses from families whose children attend schools participating in the vaccination program, 21.8% stated that the vaccination was provided in school (Table 5).

**Table 5:** Vaccination providers among total responses (total number and %)

Group	Family ped	Vaccination center	Vaccination hub	School	Private ped	Other
Participating (252)	105 (41.7%)	70 (27.8%)	15 (5.9%)	55 (21.8%)	7 (2.8%)	0 (0%)
Non-participating (1841)	920 (49.9%)	658 (35.7%)	222 (12.1%)	0 (0%)	26 (1.4%)	15 (0.8%)
Total (2093)	1025 (48.9%)	728 (34.8%)	237 (11.3%)	55 (2.6%)	33 (1.6%)	15 (0.7%)

## Discussion

This study confirms the role of schools in implementing vaccination coverage. Indeed, comparing coverage of our children aged 2–4 years vaccinated in school with those vaccinated in all settings in Italy and Lombardy, as reported in 2022–2023 vaccination coverage data of Ministry of Health, we observed a similar, if not higher, coverage in those enrolled in our school vaccination program (2–4 years 12.6% at school versus Lombardy region 14.8% versus Italy 9.2%). Since there is no Italian and Lombardy coverage data of the 5–6 aged group, a comparison could not be made [5].

In addition the school project allowed us to vaccinate for flu a significant number of children not previously vaccinated (26.2%).

It is important to consider that, we vaccinated at different times in different school areas during the flu season and each area has a different number of family pediatricians who may or may not take part in vaccinations at their ambulatory. In addition, school staff participated with different enthusiasm in schools. For example, we noticed that in the schools in zones 1 and 5, which share the same headmaster, the same vaccination rates are observed. In our view, these aspects reduce homogeneity of the study population and could represent a limitation of the study.

However, these data highlight the relevant role of schools in ensuring adequate information, through tailor-made webinar with informative material for families, other than word of mouth among parents of the same school influencing positively other students and their families to get vaccination [11, 21].

Our findings also confirm how schools play a democratic role overcoming inequalities and disparities regardless of ethnicity, socioeconomic status, and parental education level. Indeed, in our investigation, this preventive intervention was proposed to all preschoolers’ families, reaching a similar percentage in children coming from an immigrant background and Italian children (14.3% vs 11.5%).

Despite a response rate of only 19.9%, we also collected interesting information from the questionnaires that led to important considerations that will help implement the vaccination campaigns in Italy in the years to come.

We observed that 85.6% of those who responded to the questionnaire were vaccinated with the qLAIV. This formulation represents a good opportunity for the vaccination campaign: the qLAIV is, indeed, safe, non-invasive, easy to administer, well-tolerated, associated with a less biological risk for the healthcare workers and lower special waste production. As widely reported, the nasal spray formulation of the qLAIV guarantees a greater pediatric and parental compliance making an ideal formulation for the pediatric age and suitable for any setting [22].

The obtained results also show the predominant role of the family pediatrician as main provider for the vaccination. The other settings, in particular vaccination center and hub, represent an additional solution. Furthermore, 21.8% of those families participating in the vaccination program and who responded to the questionnaire were vaccinated in school, suggesting a significant role of this setting. The low response rate to the Google Form proposed to all preschools’ families weakens the data on children who participated in our vaccination programme. In this light, further studies are required to expand the literature and explore the feasibility and potential benefits of school-based vaccination campaigns.

## Conclusion

Schools are to be considered a site to inform and reach out to all families without distinction of socioeconomic status and origin, thus making it possible to offer an effective and targeted public health intervention, such as flu vaccination, equally to all.

According to our experience, in Italy, at the moment, only the cooperation between health providers and alternative settings, including schools, may expand flu vaccination coverage. In particular, schools are to be considered a place to inform and reach out to families, useful to increase vaccination coverage.

Therefore, integration among different healthcare providers may help to ensure higher vaccination coverage, according to the network model also supported by Ministry of Health recommendations for the 2023–2024 season [23].

## Data Availability

Most important elaborated data generated and analysed during the study are included in this published article. Raw data will be available on request at V. Buzzi Ospital contact Dr. Stefania Roncaglia (stefania.roncaglia@unimi.it; phone + 39.3494651694).

## Abbreviations

qLAIV: Quadrivalent Live-Attenuated Influenza Vaccine
ILI: Influenza-like illnesses
IIV: Inactivated Influenza Vaccine
